# Knowledge of myocardial infarction symptoms and perceptions of self-risk in Tanzania

**DOI:** 10.1016/j.ahj.2019.01.003

**Published:** 2019-04

**Authors:** Julian T. Hertz, Deng B. Madut, Revogatus A. Tesha, Gwamaka William, Ryan A. Simmons, Sophie W. Galson, Francis M. Sakita, Venance P. Maro, Gerald S. Bloomfield, John A. Crump, Matthew P. Rubach

**Affiliations:** a Division of Emergency Medicine, Duke University Medical Center, 2301 Erwin Rd, Durham, NC; b Department of Medicine, Duke University Medical Center, 2301 Erwin Rd, Durham, NC; c Department of Statistical Science, Duke University, PO Box 90251, Durham, NC; d Kilimanjaro Christian Medical Centre, PO Box 3010, Moshi, Tanzania; e Duke Global Health Institute, Duke University, 310 Trent Drive, Durham, NC; f Department of Emergency Medicine, Kilimanjaro Christian Medical Centre, PO Box 3010, Moshi, Tanzania; g Department of Medicine, Kilimanjaro Christian Medical Centre, PO Box 3010, Moshi, Tanzania; h Division of Cardiology, Duke University Medical Center, 2301 Erwin Rd, Durham, NC; i Otago Global Health Institute, University of Otago, PO Box 56, Dunedin, New Zealand 9054

## Abstract

**Background:**

Little is known about community knowledge of myocardial infarction symptoms and perceptions of self-risk in sub-Saharan Africa.

**Methods:**

A community survey was conducted in northern Tanzania, where the prevalence of cardiovascular risk factors is high. Households were selected randomly in a population-weighted fashion and surveys were administered to self-identified household healthcare decision-makers. Respondents were asked to list all symptoms of a heart attack and asked whether they thought they had a chance of suffering a heart attack. Associations between participant sociodemographic features and responses to these questions were assessed with Pearson's chi-squared and the Student *t* test.

**Results:**

There were 718 survey participants, with median (IQR) age 48 (32, 62) years. Of these, 115 (16.0%) were able to identify any conventional symptom of a heart attack, including 24 (3.3%) respondents who cited chest pain as a possible symptom. There was no association between ability to identify a conventional symptom and gender, level of education, socioeconomic status, urban residence, or age. Of respondents, 198 (27.6%) thought they had a chance of suffering a heart attack. Older respondents were more likely to perceive themselves to be at risk (*P* < .001), but there was no association between perception of self-risk and gender, level of education, socioeconomic status, or urban residence.

**Conclusions:**

In northern Tanzania, knowledge of myocardial infarction symptoms is poor among all segments of the population and only a minority of residents perceive themselves to be at risk of this disease. Educational interventions regarding ischemic heart disease are urgently needed.

Ischemic heart disease is the leading cause of mortality worldwide.^1^ In sub-Saharan Africa (SSA), little is known about the burden of ischemic heart disease despite the recent sharp increase in risk factors for cardiovascular disease such as obesity, hypertension, and diabetes.2., 3. A systematic review identified only a handful of studies describing the incidence or prevalence of myocardial infarction in SSA, and these were mostly performed in small populations and had methodological limitations.^4^ In Tanzania, for example, there are no rigorous incidence or prevalence data for myocardial infarction. Despite this, based on risk factor prevalence and extrapolation from other settings, the Global Burden of Disease study currently estimates ischemic heart disease to be the fourth leading cause of death in the country.^5^

The paucity of data regarding the prevalence of myocardial infarction in SSA likely has many explanations. One important factor contributing to under-detection of ischemic heart disease in the region may be the role of patient knowledge and beliefs.^6^ If patients do not recognize symptoms of acute myocardial infarctions or do not perceive themselves to be at risk for ischemic heart disease, they may not seek care that would result in an appropriate diagnosis. Thus, patient knowledge and beliefs may be important drivers of under-recognition of cardiovascular disease in SSA. Indeed, socio-cultural perceptions of cardiovascular risk factors such as diabetes and obesity have already been shown to affect healthcare utilization in the region.^7^

Little is known about community knowledge of myocardial infarction symptoms SSA, but preliminary data are discouraging.^8^ In a study of HIV-infected adults in Kenya, for example, only 1.3% of patients identified chest pain as a symptom of a heart attack.^9^ Another study performed in Cameroon found that more than 60% of patients knew that chest pain and shortness of breath were symptoms of myocardial infarction but few identified other symptoms such as jaw pain or arm pain.^10^ Even less is known about self-perception of cardiovascular risk in SSA, but data from patients with HIV suggests that many patients with risk factors do not perceive themselves to be at risk for heart disease.^9^

Aside from these sparse data, much remains to be learned about patient knowledge and beliefs regarding ischemic heart disease across SSA. In particular, given existing data about the relationship between patient beliefs and healthcare seeking behavior for other diseases in SSA,7., 11. further research is needed to understand the link between patient knowledge, perceptions, and healthcare seeking behavior for cardiovascular disease. In this study, we sought to assess community knowledge and beliefs regarding myocardial infarctions in an area of SSA with a high prevalence of cardiovascular risk factors, to identify sociodemographic features associated with such beliefs, and to describe the association between such beliefs and healthcare seeking preferences. To do so, we conducted a cross-sectional community survey in northern Tanzania.

## Methods

### Study setting

This study was performed in three districts of the Kilimanjaro Region in northern Tanzania: Moshi Urban District (population 184,289^12^), Moshi Rural District (population 466,740^12^), and Hai District (population 210,531^12^). The local prevalence of cardiovascular risk factors is high, including an estimated 28% prevalence of hypertension and 22% prevalence of glucose impairment among adults.13., 14. The predominant local tribe is Chagga.

### Participant selection

Within the study area, 60 sub-districts were selected randomly in a population-weighted fashion, with proportionate selection of urban and rural settings. Twelve points were randomly generated within each selected sub-district using Quantum Geographic Information System (QGIS, v2.18.7). The global positioning system (GPS) coordinates of each selected point was recorded and then visited on foot by the study team. The closest home to each selected point was approached for inclusion in the study. If no eligible respondent was available to participate in the survey, then the next nearest dwelling was approached. Only self-identified healthcare decision makers for the household were eligible to participate in the survey. Written informed consent was obtained from all participants.

### Survey translation

Survey questions were independently translated into Swahili and back-translated into English by two research assistants who were fluent in both languages in order to confirm fidelity to the essence of the question and to flag any potential ambiguity. Because “heart attack” can be a nebulous term, we piloted several word choice options with 15 Tanzanians with both medical and non-medical backgrounds, who were in unanimous agreement that the appropriate term in Swahili is “*mshituko wa moyo*.” This term corresponds to the biomedical English term “heart attack,” and it does not have alternate psychosocial connotations such as heartbreak, heartache, or sudden fright. The full survey was piloted with 20 Tanzanians in both urban and rural settings to ensure content clarity and fidelity prior to study initiation.

### Survey procedures

Surveys were administered in Swahili by Tanzanian field workers, using Samsung Galaxy Table A tablets (Samsung, Seoul, Korea). Surveys were designed using Open Data Kit software (ODK v1.12.2, Seattle, WA). Basic sociodemographic information was collected from all respondents, including self-reported age, gender, tribal affiliation, and religious affiliation. Respondents also were asked to report the level of education of the head of household and whether anyone in the household owned health insurance. Participants were asked to list as many symptoms of a heart attack as they could think of. Surveyors did not present participants with a list of options; participants had to name symptoms without prompting. When participants struggled to understand the biomedical concept of “symptoms,” surveyors were encouraged to explain this concept, but ultimately surveyors recorded the participant's response exactly as it was given. Respondents were also asked “Do you think you have a chance of having a heart attack?” Additionally, respondents were asked where they would go if they or another adult in their household were to experience chest pain or shortness of breath. For this question, they were asked to choose from a pick list of common types of healthcare facilities in Tanzania as well as self-treatment at home, watchful waiting, and traditional healer.

### Statistical analyses

Continuous variables are presented as means (standard deviations) or medians (ranges), and categorical variables are presented as proportions. Associations between categorical variables were analyzed with Pearson's chi-squared, and continuous variables were compared using the Student's t-test. Odds ratios and corresponding 95% confidence intervals were calculated from contingency tables to assess the magnitude of associations between categorical variables. The *t* test analyses were performed in STATA (v15.1, StataCorp, College Station, TX), all other analyses were performed in the R suite (v3.3.2, RStudio, Boston, MA). Urban residence was defined as residence within Moshi Urban District. For purposes of describing associations between level of education and other variables, education was treated as a binary variable whereby those with any form of post-primary education (secondary school, post-secondary school) were compared to all other respondents. Conventional myocardial infarction symptoms were defined a priori as chest pain, shortness of breath, nausea, dizziness, epigastric pain, diaphoresis, jaw pain, and arm pain. A socioeconomic status score was derived via principal component analysis^15^ from nine binary variables: post-primary education, presence of electricity in the home, health insurance coverage, home floor material, ownership of a bank account, ownership of a car, ownership of a TV, ownership of a refrigerator, and presence of a flush toilet in the home.

### Ethical review

This study received ethics approval from the Duke Health Institutional Review Board, the Kilimanjaro Christian Medical Centre Research Ethics Committee, and the Tanzania National Institutes for Medical Research Ethics Coordinating Committee.

### Funding

This study was funded by Bill & Melinda Gates Foundation grant OPP1158210. The authors are solely responsible for the design and conduct of this study, all study analyses, the drafting and editing of the paper and its final contents.

## Results

A total of 718 persons participated in the survey, and their sociodemographic features are summarized in Table I. The median (IQR) age of participants was 48 (32, 62) years, and 485 (67.5%) of respondents were female. The majority of respondents lived in rural areas (563, 78.4%) and did not have post-primary education (537, 74.8%).

**Table I:** Sociodemographic features of household survey respondents, Moshi Urban, Moshi Rural, and Hai districts, 2018 (N = 718)

	n	(%)
Female	485	(67.5)
Urban residence	155	(21.6)
Education		
None	40	(5.6)
Primary	497	(69.2)
Secondary	132	(18.4)
Post-Secondary	49	(6.8)
Have health insurance	230	(32.0)
Religion		
Christian	584	(81.3)
Muslim	115	(16.0)
Other	19	(2.6)
Chagga tribe	535	(74.5)

	Median	(Range)

Age, years	48	(17, 99)
Household size, number of persons	4	(1, 13)
SES score	0.29	(0, 1.01)

*SES*, socioeconomic status.

The symptoms of myocardial infarctions identified by respondents are presented in Table II. The majority of participants (403, 56.1%) were unable to name any symptom of a heart attack. The most commonly mentioned symptoms of a heart attack were feelings of worry, sadness, or anger (93, 13.0%) and headache (52, 7.1%). One hundred fifteen (16.0%) respondents were able to identify at least one conventional symptom of a myocardial infarction. The most commonly identified conventional symptom was sweating, mentioned by 36 (5.0%) participants. Chest pain was cited as a symptom of a heart attack by 24 (3.3%) participants.

**Table II:** Symptoms of a ‘heart attack’ cited by residents of northern Tanzania, 2018 (N = 718)

Symptom	Number of respondents (%)
Don't know any	403 (56.1)
Feelings of worry, sadness, or anger	93 (13.0)
Headache	52 (7.1)
Palpitations	43 (6.0)
Sweating	36 (5.0)
Shortness of breath	35 (4.9)
Dizziness	33 (4.6)
Chest pain	24 (3.3)
Fever	20 (2.8)
High blood pressure	19 (2.6)
Unilateral paralysis	18 (2.5)
Loss of consciousness	12 (1.7)
Fatigue	10 (1.4)
Confusion	5 (0.7)
Jaw or arm pain	4 (0.6)
Nausea	3 (0.4)
Leg swelling	3 (0.4)
Epigastric pain	1 (0.1)
Others	17 (2.4)

Table III compares the sociodemographic features of respondents who were able to identify at least one conventional myocardial infarction symptom versus those who were not. There were no statistically significant associations between ability to identify a conventional symptom and education, urban residence, age, or socioeconomic status. There were also no significant associations between ability to identify a convention symptom and preference for a hospital for symptoms of chest pain or shortness of breath.

**Table III:** Characteristics of participants who identified any conventional myocardial infarction symptom versus those who did not, northern Tanzania, 2018 (N = 718)⁎

	Able to name a conventional symptom, n (%) (N = 115)	Unable to name a conventional symptom, n(%) (N = 603)	OR (95% CI)	*P* value
Female	78 (67.8%)	407 (67.5%)	1.02 (0.66, 1.56)	.945
Urban residence	25 (21.7%)	130 (21.6%)	1.01 (0.62, 1.64)	.966
Post-primary education	23 (20.0%)	158 (26.2%)	0.70 (0.43, 1.15)	.160
Health insurance	38 (33.0%)	192 (31.8%)	1.06 (0.69, 1.62)	.800
Chagga tribe	85 (73.9%)	450 (74.6%)	0.96 (0.61, 1.52)	.872
Hospital preferred facility for chest pain	47 (40.9%)	230 (38.1%)	1.12 (0.75, 1.68)	.582
Hospital preferred facility for shortness of breath	92 (80.0%)	430 (71.3%)	1.61 (0.99, 2.63)	.055

	Able to name a conventional symptom, mean (SD) (N = 115)	Unable to name a conventional symptom, mean (SD) (N = 603)		*P* value

Age, years	48.6 (18.5)	48.0 (18.0)		.732
SES score	0.32 (0.28)	0.35 (0.30)		.239

*SES*, Socioeconomic status.

⁎ *P* < .05.

A total of 198 (27.6%) of respondents stated they thought they had a chance of suffering a heart attack. Of the remaining respondents, 310 (43.2%) did not think they had any chance of having a heart attack and 210 (29.2%) did not know whether they were at risk. Table IV compares the sociodemographic features of participants who perceived themselves to be at risk for a heart attack versus other respondents. Compared to others, respondents who perceived themselves to be at risk for a heart attack were more likely to be older (mean age 52.8 vs 46.3 years, *P* < .001). There were no differences in gender, education, or socioeconomic status between the two groups. Self-perceived risk was not associated with increased likelihood of preferring a hospital for chest pain or shortness of breath.

**Table IV:** Characteristics of respondents who felt they were at risk of heart attacks versus those who did not perceive any self-risk or were unsure, northern Tanzania, 2018 (N = 718)

	Perceived themselves to be at risk, n (%) (N = 198)	Did not perceive themselves to be at risk or were unsure, n (%) (N = 520)	OR (95% CI)	*P* value
Female	134 (67.7%)	351 (67.5%)	1.01 (0.71, 1.43)	.964
Urban	42 (21.2%)	113 (21.7%)	0.97 (0.65, 1.45)	.880
Post-primary education	42 (21.2%)	139 (26.7%)	0.74 (0.50, 1.10)	.128
Health insurance	64 (32.3%)	166 (31.9%)	1.02 (0.72, 1.45)	.918
Identified a conventional symptom of myocardial infarction	32 (16.2%)	83 (16.0%)	1.01 (0.65, 1.58)	.948
Chagga tribe	151 (76.3%)	384 (73.8%)	1.14 (0.78, 1.67)	.392
Hospital preferred facility for chest pain	68 (34.3%)	209 (40.2%)	0.78 (0.55, 1.10)	.150
Hospital preferred facility for shortness of breath	134 (67.7%)	388 (74.6%)	0.71 (0.50, 1.02)	.062

	Perceived themselves to be at risk, mean (SD) (N = 198)	Did not perceive themselves to be at risk or were unsure, mean (SD) (N = 520)		*P* value
Age, years	52.8 (17.1)	46.3 (18.2)		<.001⁎
SES score	0.31 (0.27)	0.36 (0.30)		.052

*SES*, Socioeconomic status.

⁎ *P* < .05.

## Discussion

This study is among the first to examine community knowledge of myocardial infarction symptoms and self-perceived risk in SSA. In a community with high prevalence of risk factors,13., 14. only a minority of residents perceived themselves to be at risk for suffering a heart attack. Furthermore, knowledge of myocardial infarction symptoms was limited. These findings underscore a grave need for educational programming to improve community awareness of ischemic heart disease.

The vast majority of respondents were unable to name any conventional symptom of a myocardial infarction, consistent with the results of the few other studies regarding knowledge of myocardial infarction symptoms in SSA.8., 9. Knowledge of symptoms was especially poor in our study setting, with only 16% of participants able to name a single symptom, the lowest proportion reported in SSA to date.8., 10. Lack of knowledge of the symptoms of this life-threatening condition was not confined to a single segment of the population; low levels of knowledge were observed across all ages, genders, tribes, education levels, and socioeconomic strata. Thus, the need for community educational programming in northern Tanzania is particularly acute, especially given the high local prevalence of risk factors.13., 14. In recent years, the Tanzanian Ministry of Health, the Tanzanian Cardiac Society, and others have increased efforts to educate the community about symptoms of heart disease via media programming which have emphasized chest pain, shortness of breath, and dizziness as potential warning signs.16., 17., 18. The results presented here suggest that such efforts have not yet resulted widespread community recognition of the symptoms of myocardial infarction in northern Tanzania.

Less than a third of participants in this study felt that they were at any risk of having a myocardial infarction despite the high local prevalence of cardiovascular risk factors.13., 14. Older respondents were more likely to consider themselves to be at risk, but otherwise lack of self-perception of risk was not limited to any specific gender, education level, socioeconomic stratum, urban or rural setting, or tribe. Thus, efforts by clinicians, public health officials, and community leaders are needed to emphasize personal risk of cardiovascular disease in educational programming. These efforts would be more effective if informed by data regarding local burden of disease, but such data are presently lacking in Tanzania. Therefore, establishing the local prevalence of ischemic heart disease is essential to formulating a public health response to the observed low levels of knowledge of myocardial infarction symptoms and perception of self-risk.

In this population, knowledge of myocardial infarction symptoms and perception of self-risk were not associated with preference for hospital care for common symptoms of cardiovascular disease such as chest pain and shortness of breath. This finding is concerning because in northern Tanzania, the capacity for electrocardiogram testing and cardiac enzyme testing is limited to hospitals. Therefore, educational interventions regarding myocardial infarction ought to include information about appropriate care seeking for myocardial infarction symptoms rather than just recognition of such symptoms or emphasizing personal cardiovascular risk. Previous research in Tanzania found that among patients admitted to referral hospitals with severe febrile illness, seeking care at multiple lower level health facilities and experiencing delays in accessing referral hospitals was associated with increased mortality.^19^ It is unknown whether such patterns of delays within the healthcare system are also associated with increased mortality for cardiovascular diseases in Tanzania.

Our study had several limitations. In an attempt to survey those whose opinions guide actual healthcare utilization, only self-identified healthcare decision makers were eligible for inclusion in this study. Thus, the study sample may not be representative of adults in northern Tanzania as a whole. Furthermore, the survey was conducted in homes during daytime hours, which may have resulted in under-representation of males and individuals with certain occupations. Moreover, when asked to list symptoms of myocardial infarction, participants were not given a list of options to choose from, in order to avoid biasing them towards any specific set of answers. However, it is possible that more respondents would have identified a conventional heart attack symptom if they had been given a picklist. Finally, information about individual respondents' cardiovascular risk profiles was not collected which would have allowed for a more nuanced analysis of perceptions of self-risk. Nonetheless, given the known high local burden of risk factors like hypertension, the low proportion of adult respondents in this study who felt they were at risk of a heart attack, and the lack of association between age and perception of self-risk observed in our study, there is clearly a need for increased awareness of cardiovascular risk in northern Tanzania.

In conclusion, knowledge of myocardial infarction symptoms was low in northern Tanzania, and few residents perceived themselves to be at risk for heart attacks. More research is needed to determine the local prevalence of ischemic heart disease and to develop effective educational interventions regarding this life-threatening condition.
